# Self-Shielding for the ZAP-X®: Revised Characterization and Evaluation

**DOI:** 10.7759/cureus.13660

**Published:** 2021-03-02

**Authors:** Georg A Weidlich, M. Bret Schneider, Vilim Simcic, Zander Oostman, John R Adler

**Affiliations:** 1 Radiation Oncology, National Medical Physics and Dosimetry Company, Palo Alto, USA; 2 Research and Development, Zap Surgical Systems, San Carlos, USA; 3 Radiation Oncology, Stanford University Medical Center, Stanford, USA; 4 Neurosurgery, Stanford University School of Medicine, Stanford, USA

**Keywords:** self-shielding, shielding analysis, radiation safety, intracranial radiosurgery

## Abstract

The ZAP-X® is a newly designed, self-contained, and first-of-its-kind self-shielded therapeutic radiation therapy device dedicated to brain and head and neck stereotactic radiosurgery (SRS). By using an S-band linear accelerator (linac) and employing integrated minimal but sufficient shielding, the ZAP-X does not typically require a radiation bunker. At the same time, the self-shielded features of the ZAP-X are designed for more consistency of radiation protection, reducing the risk to radiation workers and others potentially exposed from a poorly designed or constructed radiotherapy vault.

This study postulates that a radiosurgical system can be self-shielded, such that it produces radiation exposure levels deemed safe to the public while operating under a full clinical workload. The goal of self-shielding is achieved under all but the most exceptional clinical conditions.

This work is intended to serve as guidance for the radiation safety evaluations of future ZAP-X treatment operations, following local or regional applicable regulatory requirements, and utilizing the unique provision of all or most of the required shielding material as an integral part of the device.

## Introduction

The ZAP-X® system is a dedicated, self-contained, and self-shielded radiosurgery system developed and manufactured by ZAP Surgical Systems, Inc., of San Carlos, California. Utilizing an S-band linear accelerator with a 3.0-MV accelerating potential, this device is designed specifically for stereotactic radiosurgical (SRS) ablation of intracranial and head and neck lesions [1].

The different structural elements of the ZAP-X are arrayed to provide the shielding effect that typically is established by the walls, ceiling, and floor of a radiotherapy vault [2,3]. Most components needed to produce the therapeutic beam, such as the radiofrequency power source, waveguide system, beam triggering electronics, and a dedicated beam stop, are mounted on or integrated into the primary spherical supporting structure. Furthermore, the patient (who is positioned supine) is enclosed by yet additional scatter shielding consisting of a rotatable iron shell and a shielded, pneumatically elevated door at the foot of the treatment table. By being mounted onto a shielded treatment sphere with dual-axes of independent rotation, the treatment beam from the linear accelerator can be isocentrically positioned across a solid angle of over 2π steradian, as necessitated for cranial SRS.

Image guidance is provided by planar image acquisition and image-to-image matching with digitally reconstructed radiographs (DRRs) calculated from the treatment planning system (TPS).

This revised study will demonstrate that the ZAP-X is self-shielded according to the National Council on Radiation Protection (NCRP) and the Nuclear Regulatory Commission (NRC), as well as European, Asian, and Japanese standards while operating under a heavy clinical workload [2,3]. As the ZAP-X has been in clinical use for over two years, a considerable amount of clinical data has been accumulated describing the typical use of the system, thereby providing a refined set of workload parameters and an accurate shielding evaluation.

## Materials and methods

The entire ZAP-X system, including the shielded patient spherical chamber, the treatment table enclosure, and a rotating beam stop, was designed to provide an amount of shielding effect that would be provided by the walls, ceiling, and floor of a radiotherapy treatment vault.

The goals of the self-shielded ZAP-X system are to: (i) Provide shielding to treatment personnel and members of the public outside a 1-m safety zone from the perimeter of the ZAP-X system to 1 milliSievert (mSv)/year. This limit is the accepted value for non-radiation personnel and stipulated by the NCRP [2,3], being 50 times lower than the allowable limit for radiation workers (50 mSv). (ii) Provide shielding at any one point along the above-described perimeter line that results in a composite instantaneous exposure rate of no more than 2.0 milliroentgens per hour (mR/h). One should note that as any location of the gimbal combination rotates during treatment delivery, this relatively large instantaneous exposure rate is never measurable in one direction for an extended period of time. (iii) Provide all required shielding typically present in the facility shielding.

The shielding material is composed mainly of ductile iron supplemented by high Z materials consisting of steel, lead, or tungsten alloys. The placement of radiation shielding, as well as the thickness and materials to be used, was determined using radiation transport computational simulations based on the Monte Carlo dose algorithm [4-6]. Primary radiation, as well as leakage and scatter radiation, was taken into account, and the required shield thicknesses were determined at a number of points on this sphere and designed according to the Radiation Protection Guidelines defined in NCRP reports 116 and 151 [2,3].

**Figure 1:**
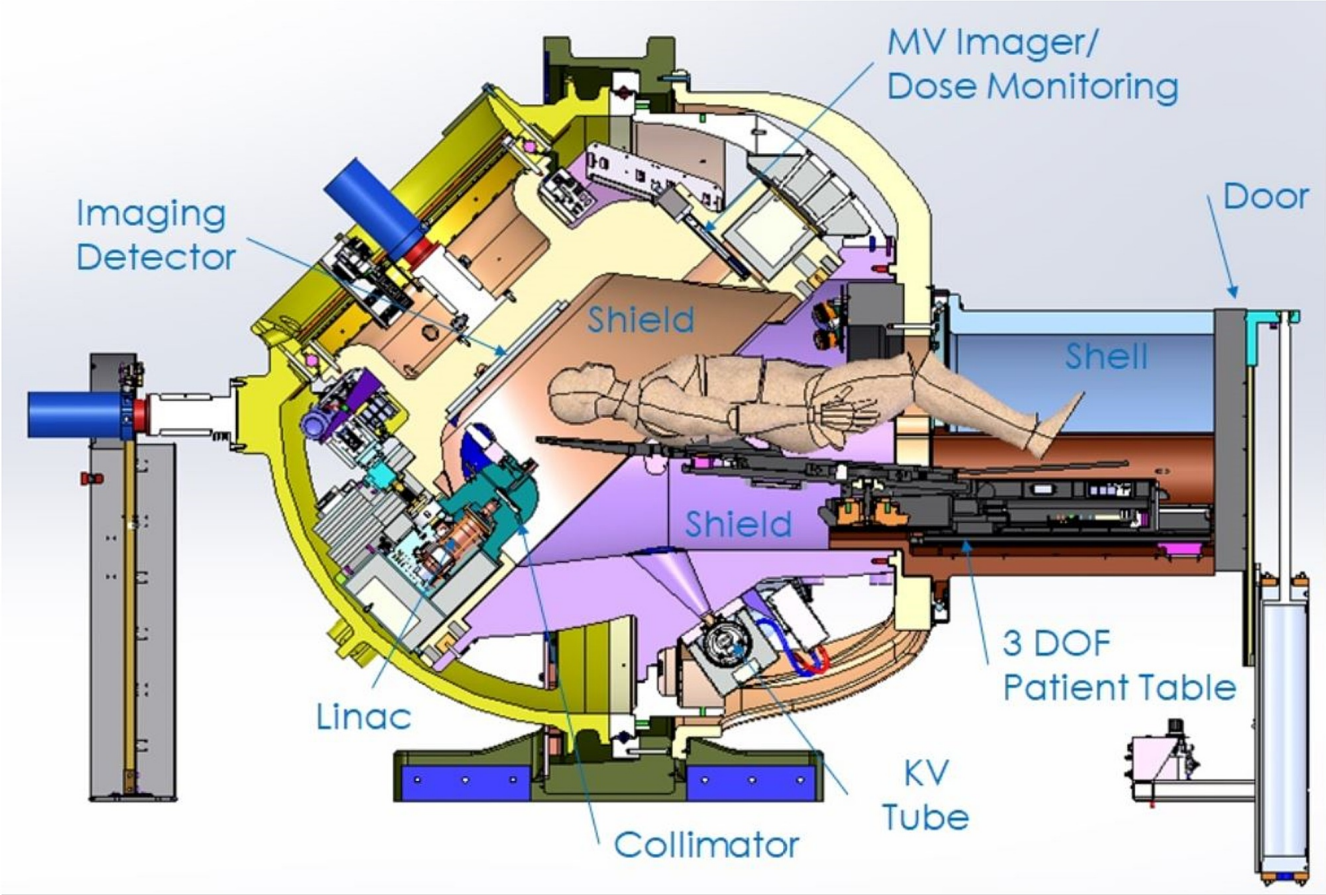
The ZAP-X: a cross-sectional view of the patient in the treatment position. DoF: degrees of freedom, KV: kilovolt, MV: megavoltage.

The cross-sectional view and room’s eye view of the ZAP-X are shown in Figure 1.

The focus of this work was the systematic evaluation of the prospective ZAP-X shielding requirements in order to provide safe clinical operations. Parameters determining such evaluations are the patient workload (W), the duty cycle (DC), use factor (U), the occupancy factor (T), and the maximum radiation leakage instantaneous dose rate (IDR) produced by the system at the 1 m safety distance from the system. No access is granted inside this safety zone distance.

Typical treatments for intracranial radiosurgery include a variety of clinical indications, such as solitary brain metastases, meningiomas, schwannomas, glioblastomas, trigeminal neuralgia, acoustic neuromas, and arteriovenous malformations. Treatments for such indications are delivered with one to five treatment fractions with prescription doses per fraction ranging from 5 grays (Gy) to 75 Gy.

The primary beam stop of the ZAP-X that remains diametrically opposed to the radiation source during rotation is extremely well-shielded with more than 5.5 tenth value layers (TVLs) of shielding. Such shielding provides less than 0.00032% of radiation transmission, which is more than two orders of magnitude lower than other secondary radiation sources, such as X-ray leakage and patient scatter. Beyond the typical 3 TVLs for a beam stop, an additional 2.5 TVLs were applied to suppress radiation transmission well below the customary limit of 0.1% of primary radiation. As a typical number of beams applied per treatment fraction is 33 to 100, the use factor for a specific primary beam direction is extremely small at approximately 0.03 to 0.01, respectively, and the composite contribution of the primary beam at any one point is, therefore, negligible.

The intensity-modulated radiation therapy (IMRT) factor for complex ZAP-X treatments can range from 5 to 18 and most of the radiation that will contribute to external leakage is secondary radiation caused by patient scatter and X-ray target leakage radiation. This secondary radiation is directly determined by the amount of ‘beam on’ time or the system monitor units (MUs) delivered. As the overwhelming shielding requirements originate from secondary radiation sources, the basis for the shielding calculation was chosen to be MU as the clinical workload and not the typically applied dose in Gy.

Historic clinical data and prospective patient treatment projections suggest a mean prescription dose of 1,350 cGy per treatment fraction given, averaged over all treatment courses. As an example, for 75 treatments delivered per week, this would correspond to a workload of 101,250 cGy per week or 1012.5 Gy per week. While these data will not be directly used for the quantitative evaluation of the ZAP-X shielding, they are mentioned here to provide the necessary perspective from historically applied shielding evaluation techniques.

Clinically delivered ZAP-X treatments were tracked via a central database and the first 85 patients were taken into account. All radiosurgical treatments were planned using the ZAP-X TPS [7]. Patients were treated with a mean of 6.5 isocenters per treatment and a mean of 7,021 MU per treatment fraction. To allow more complexity in treatments, as well as radiation oncology and neurosurgery-influenced programs, 12,000 MU per treatment fraction was assumed as the upper limit for treatments regarding prospective shielding evaluations. The resulting IMRT factor would be 12,000 MU/1,350 cGy = 8.889 based on the dose of 1,350 cGy delivered per fraction. The mean number of treatment fractions per patient was 2.8. This number is naturally dependent on the type of medical practice, specifically, if neurosurgery or radiation oncology influence prevails in the practice being evaluated.

Assuming five treatment days per week and 50 treatment weeks per year, the total annual workload (W) expressed in MU and treatments (Tx) per day can be calculated as:

 W = 12,000 MU × Tx × 5 d/week × 50 weeks/y = 3.0 × 10^6^ MU/y × Tx (1)

The DC expressing the percentage of time that the Linac is energized for the described clinical treatments can then be calculated by dividing the workload in MU by the machine dose rate of 1,500 MU/min = 90,000 MU/h and the number of working hours in one year (2000 h/y) to be:

 DC = (3.0 × 10^6^ MU/y × Tx) / (90,000 MU/h × 2,000 h/y) = 0.01667 × Tx (2)

Additional parameters entering into the shielding considerations are the use factor (U) and the occupancy factor (T). As U is mainly applied for the consideration of primary shielding barriers and our main concern are secondary sources of radiation, such as X-ray target radiation leakage and patient scatter, we assume U = 1.0. Additionally, we apply a composite IDR which is empirically determined from a typical ZAP-X treatment.

The shielding design of the ZAP-X is based on providing shielding material to allow an annual dose of no more than 1.0 mSv for a given patient workload at any one point along a perimeter line with a distance of 1 m from the surface of the ZAP-X. In order to achieve that, a number of shielding upgrades were implemented including an external shield on the back of the linear accelerator, and the system was evaluated after the upgrade in early 2021. The area inside this perimeter line is not accessible since a laser surveillance scanning system will interrupt any radiation and system motion if entered by a person. As this 1 m safety perimeter line is inside the treatment room and only occasional visitors (being members of the public, such as family members, referring physicians, or hospital staff) will be present in the room during treatment delivery, an occupancy factor of T = 1/16 = 0.0625 is assigned.

The IDR was determined from measurements along the 1 m safety perimeter line and will be presented, analyzed, and evaluated in detail below.

Radiation exposure rates as a function of beam position

**Figure 2:**
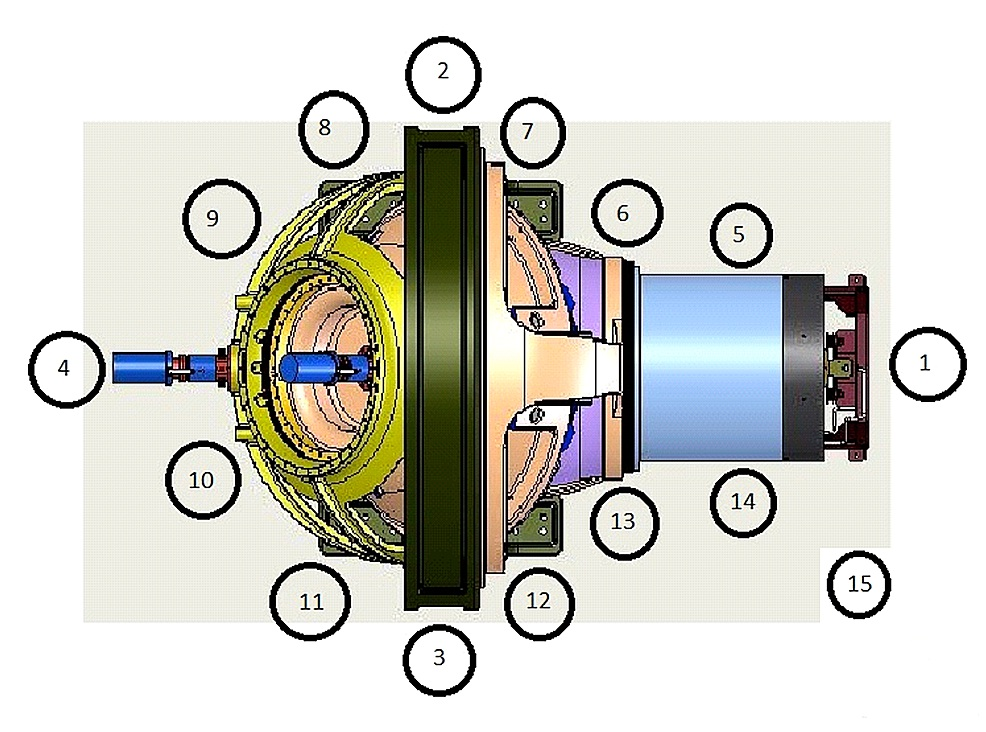
Measurement stations 1 through 15 along the 1 m perimeter line. Stations are indicated as circled numbers.

Based on the above initial design assumptions, radiation levels at numerous points along the 1 m safety perimeter line from the ZAP-X system were measured for all cardinal beam positions at a height of 1.2 m above floor level. Instantaneous exposure rates, as well as time-integrated measurements of exposures throughout the entire treatment, were determined for 14 equidistant stations along the perimeter line of the ZAP-X and the control station using a Victoreen Model 451 survey meter (Fluke Biomedical, Everett, WA). Figure 2 illustrates the position of measurement locations, as viewed from above.

Radiation accumulative exposure

Using the reference treatment of 12,000 MU, a treatment plan was generated on the ZAP-X TPS system and delivered [7]. The accumulative exposure was measured at the same 14 measurement stations along the 1 m safety perimeter line. One additional measurement location atop the system was placed at 1 m above the surface of the treatment sphere (atop the sphere).

## Results

Instantaneous exposure rate measurements

Instantaneous exposure rates are summarized in Table 1, showing composite results at each of the measurement positions for each beam position.

**Table 1:** Summary of instantaneous exposure rates (mR/h) for various gantry positions. The gantry position is as indicated. Composite exposure rates in mR/h are based on the mean value for each of the five tested gantry angles. A quality factor of 1.0 was applied for the 3 MV photon radiation of the ZAP-X. Maximum of composites: 1.56 mR/h; composite for control console: 0.05 mR/h AP: anteroposterior, Bkg: background, LT LAT: left lateral, mR/h: milliroentgens per hour, MV: megavoltage, PA: posteroanterior, RT LAT: right lateral.

Position	Description of Stations	Home	AP	RT LAT	LT LAT	PA	Composite (mR/h)
1	Foot End Door	3.00	0.37	2.88	1.44	0.09	1.560
2	Right Main Gantry	0.06	0.05	0.03	0.22	0.04	0.080
3	Left Main Gantry	0.11	0.07	0.11	Bkg	0.08	0.074
4	Head End of System	1.80	0.28	1.80	0.14	0.09	0.822
5	Table Right	0.19	0.25	0.71	0.14	0.17	0.292
6	Table – Orbit Right	0.17	0.42	0.91	0.19	0.15	0.368
7	Right Gantry	0.04	0.45	0.65	0.19	0.15	0.296
8	Right Gantry	1.72	0.22	0.11	1.47	0.24	0.752
9	Right – Head	1.80	0.77	0.14	2.70	0.23	1.128
10	Left – Head	2.80	0.21	1.35	0.03	0.29	0.936
11	Left Gantry	1.72	0.22	1.02	0.14	0.12	0.644
12	Left Gantry	0.10	0.78	0.06	0.33	0.72	0.398
13	Table – Orbit Left	0.18	1.41	0.08	0.64	0.89	0.640
14	Table Left	0.19	0.43	0.09	0.63	0.36	0.340
15	Control Console	Bkg	0.04	0.02	0.15	0.04	0.050

Determination of the Maximum Number of Patient Treatments at the 1 M Perimeter Line (Public Exposure)

A composite exposure rate value for each of the 15 measurement stations was calculated; the maximum for any of the stations is 1.56 mR/h. Additionally, several high exposure rate points outside the sphere of the ZAP-X were found with a maximum value of 13 mR/h. These points were detected between the described measurement stations and originated from the penetrations of the Linac and X-ray tube through the shielding sphere. Due to the beam directions and used cardinal gantry angles, this leakage of radiation is not detectable during a typical survey as indicated in Table 1.

These higher intensity beams were evaluated individually using the model for a treatment with a maximum number of beams. The probability of any such beams intercepting the occupiable space was determined along the 1 m perimeter line between the 150 cm and 80 cm height from the floor level, representing the typical human thyroid and gonads location. The probability of a hot spot from the rear of the ZAP-X was determined to be 31% and 7.6% from the axial shield direction. For the worst-case scenario, the 13 mR/h beam would, therefore, add 13 mR/h × 0.31 = 4.03 mR/h to the combined 15 measurement stations or 4.03 mR/h × 1/15 = 0.269 mR/h to each station. Therefore, the maximum composite exposure rate of 1.560 mR/h increased by 0.269 mR/h to 1.829 mR/h. The resulting adjusted maximum composite exposure rate was assumed to be less than 2.0 mR/h. For calculation purposes, this is equivalent to an IDR of 0.02 mSv/h.

The following relationship can be established incorporating the maximum allowable annual dose of 1.0 mSv/y for the public that will have access to the treatment room during operations:

 IDR × U × T × DC × 2,000 h/y < 1.0 mSv/y or 

 0.02 mSv/h × 1.0 × 0.0625 × 0.01667 × Tx × 2000 h/y < 1 mSv/y or (3)

 Tx < 24.0 treatments per day.

Determination of Maximum Number of Patient Treatments at the Control Console

The composite exposure rate for the control console was determined to be 0.05 mR/h. Additionally, the known high exposure rate points with 13 mR/h which occur for every treatment, as described above, were followed and their contribution to the control console between a height of 120 cm and 50 cm height from the floor level was determined, corresponding to the anatomy of a sitting person at the console. The probability of a high-intensity beam intercepting the control console was determined to be 4.4% for a plan with all possible beams turned on. However, this beam will not be directed towards the control console for practical clinical treatments due to beam clearance issues. For the worst-case scenario, the 13 mR/h beams would, therefore, add 0.572 mR/h at the scanner boundary and 0.572 mR/h x [(2.03 m)^2^/(3.15 m)^2^] = 0.238 mR/h to the exposure of the two seats at the control console or 0.119 mR/h for the one seat position. This component of the exposure rate was added and the adjusted composite exposure rate for the console was determined to be 0.169 mR/h. The resulting adjusted maximum composite exposure rate was assumed to be less than 0.2 mR/h. For calculation purposes, this is equivalent to an IDR of 0.002 mSv/h.

Using an occupancy factor of T = 1.0, the following relationship can be established incorporating the maximum allowable annual dose of 1.0 mSv/y for the public that will have access to the treatment room during operations:

IDR × U × T × DC × 2,000 h/y < 1.0 mSv/y or

0.002 mSv/h × 1.0 × 1.0 × 0.01667 × Tx × 2000 h/y < 1 mSv/y or (4)

Tx < 15.0 treatments per day.

As the control console represents the more stringent requirement, this condition shall be adopted to establish the maximum number of patients treatable.

Accumulative exposure measurements

For the treatment plan-produced radiation dose, the accumulative measurements are summarized in Table 2.

**Table 2:** Summary of accumulative exposure measurements. µR: microroentgen.

Station	Exposure (µR)
1	60
2	10
3	10
4	58
5	64
6	16
7	8
8	48
9	61
10	59
11	46
12	54
13	0
14	58
Atop sphere	106

The calculated maximum annual exposure was detected at the station above the ZAP-X and measured 106 µR (microRoentgen). The maximum allowable workload of 15 treatments per day or 3,750 treatments per year, an occupancy factor of 0.25, and 100 mR/mSv, will result in an expected maximum annual dose of 0.994 mSv which is less than the annual limit of 1 mSv/y and confirms the findings from the exposure rate measurements.

For the most limiting conditions at the treatment console, a maximum of 15.0 daily treatment fractions or 3,750 annual treatment fractions can be safely delivered. As typical treatments require a minimum of 30-minute time slots in clinical operation, 15.0 treatments per day will constitute a single shift/full-time clinical workday (7.5 h of work, 30 min break). Assuming 2.8 treatment fractions per patient, an annual maximum of 1339.3 patients would be allowed. Only the most extreme clinical demands are expected to exceed such a workload.

Combining equations (1), (2), and (4), the product of the number of MU per treatment times the treatments per day can be expressed as:

#MU × Tx < 180,000 (5)

This relationship will allow future users to determine if, for a given workload in a specific clinical setting with known treatment complexity and MUs per treatment, the self-shielding characteristics will remain preserved. One should note that Tx does not depend on the number of fractions per treatment course. For example, in an exclusive neurosurgery caseload with all treatment courses delivered in a single fraction and very complex treatments of 20,000 MUs/treatment, Tx would result in nine (treatments per day) and with this workload, the system will remain self-shielded.

**Figure 3:**
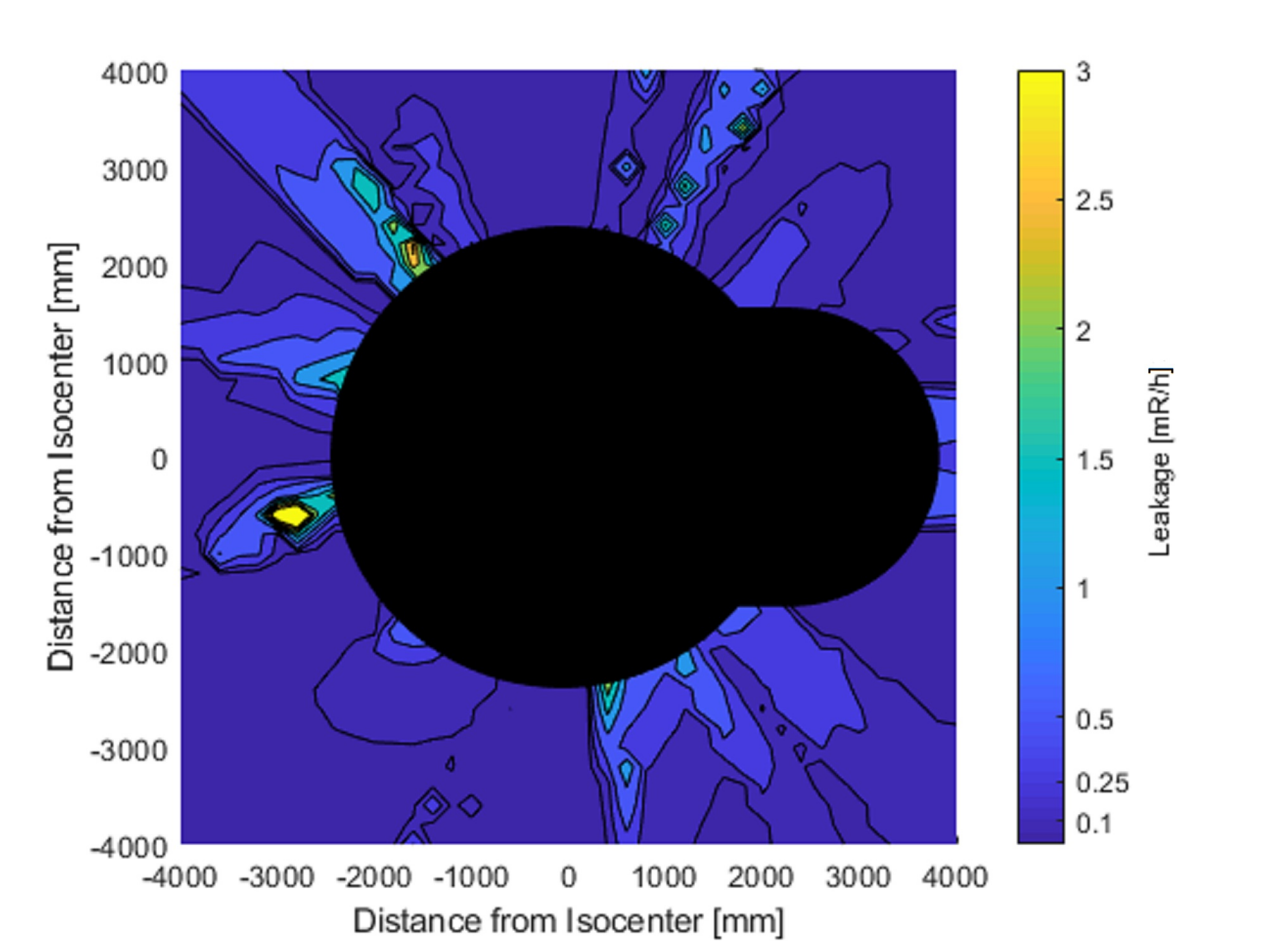
Two-dimensional radiation leakage matrix in a horizontal plane at isocenter height. The origin is located at the isocenter. mR/h: milliroentgens per hour, mm: millimeter.

As most of the ZAP-X system acceptance testing will be performed with the beam pointing straight down, a two-dimensional (2D) radiation leakage matrix was generated to represent the radiation propagation in a horizontal plane at isocenter height. This matrix is shown in Figure 3.

## Discussion

Using the example of 12,000 MU per treatment, for a maximum workload of 15 treatments per day, the above exposure rate measurements result in an annual dose equivalent of 1.0 mSv/year, which is the generally allowable maximum radiation dose for the public [2,3]. In light of this observation, the ZAP-X system, under the specified workload, would allow unrestricted access for non-radiation workers outside a 1 m system perimeter and to all areas on the floor above the ZAP-X treatment room. Therefore, the design of the ZAP-X system satisfies all shielding requirements and no shielded treatment room is required.

Due to the self-shielding materials provided as an integral part of the ZAP-X system, the resulting radiation leakage will remain below the allowable annual dose levels for the public without the provision of a treatment bunker. Therefore, for the defined workload, the annual dose delivered to persons outside the safety perimeter will not exceed 2% of the dose allowable for radiation workers (50 mSv). Since the shielding is provided as part of the system design, no additional effort or time will be required for the design and construction of facility-based shielding.

The acceptable workload can be increased if the access to the treatment room is restricted, the area inside the treatment room is reclassified to be occupationally exposed, or additional shielding in the walls of the treatment room is provided.

The revised analysis focuses on establishing a maximum workload as defined by the treatment monitor units and the number of treatments per day that will allow future users to verify the adequacy of facility shielding. The described analysis will assist future users to verify that, for a planned workload, the system will be self-shielded.

## Conclusions

The analysis presented is an *a priori* derivation of the ZAP-X maximum allowable patient census for a given type of practice setting and complexity of treatment. For the most realistic combinations of the patient census and treatment complexity, the ZAP-X system is considered self-shielded by NRC and NCRP guidelines, and, therefore, most clinical user sites will not require a radiation treatment bunker. Future users of this technology can readily apply the findings of this analysis to verify the ZAP-X self-shielding characteristic for their institution.
